# Assessing the adherence of large language models to clinical practice guidelines in Chinese medicine: a content analysis

**DOI:** 10.3389/fphar.2025.1649041

**Published:** 2025-07-25

**Authors:** Weilong Zhao, Honghao Lai, Bei Pan, Jiajie Huang, Danni Xia, Chunyang Bai, Jiayi Liu, Jianing Liu, Yinghui Jin, Hongcai Shang, Jianping Liu, Nannan Shi, Jie Liu, Yaolong Chen, Janne Estill, Long Ge

**Affiliations:** ^1^ Department of Health Policy and Management, School of Public Health, Lanzhou University, Lanzhou, China; ^2^ Evidence-Based Medicine Center, School of Basic Medical Sciences, Lanzhou University, Lanzhou, China; ^3^ College of Nursing, Gansu University of Chinese Medicine, Lanzhou, China; ^4^ School of Nursing, Southern Medical University, Guangzhou, China; ^5^ Institute of Global Health, University of Geneva, Geneva, Switzerland; ^6^ Center for Evidence-Based and Translational Medicine, Zhongnan Hospital of Wuhan University, Wuhan, China; ^7^ Key Laboratory of Chinese Internal Medicine of Ministry of Education and Dongfang Hospital, Beijing University of Chinese Medicine, Beijing, China; ^8^ Evidence-Based Medicine Center, Beijing University of Chinese Medicine, Beijing, China; ^9^ Institute of Basic Research in Clinical Medicine, China Academy of Chinese Medical Sciences, Beijing, China; ^10^ Department of Oncology, Guang’anmen Hospital, China Academy of Chinese Medical Sciences, Beijing, China; ^11^ Research Unit of Evidence-Based Evaluation and Guidelines (2021RU017), Chinese Academy of Medical Sciences, School of Basic Medical Sciences, Lanzhou University, Lanzhou, China; ^12^ Key Laboratory of Evidence Based Medicine of Gansu Province, Lanzhou, China; ^13^ Institute of Health Data Science, Lanzhou University, Lanzhou, China; ^14^ World Health Organization Collaborating Centre for Guideline Implementation and Knowledge Translation, Lanzhou, China

**Keywords:** Chinese medicine, large language model, comparison, clinical practice guideline, knowledge acquisition

## Abstract

**Objective:**

Whether large language models (LLMs) can effectively facilitate CM knowledge acquisition remains uncertain. This study aims to assess the adherence of LLMs to Clinical Practice Guidelines (CPGs) in CM.

**Methods:**

This cross-sectional study randomly selected ten CPGs in CM and constructed 150 questions across three categories: medication based on differential diagnosis (MDD), specific prescription consultation (SPC), and CM theory analysis (CTA). Eight LLMs (GPT-4o, Claude-3.5 Sonnet, Moonshot-v1, ChatGLM-4, DeepSeek-v3, DeepSeek-r1, Claude-4 sonnet, and Claude-4 sonnet thinking) were evaluated using both English and Chinese queries. The main evaluation metrics included accuracy, readability, and use of safety disclaimers.

**Results:**

Overall, DeepSeek-v3 and DeepSeek-r1 demonstrated superior performance in both English (median 5.00, interquartile range (IQR) 4.00–5.00 vs. median 5.00, IQR 3.70–5.00) and Chinese (both median 5.00, IQR 4.30–5.00), significantly outperforming all other models. All models achieved significantly higher accuracy in Chinese versus English responses (all p < 0.05). Significant variations in accuracy were observed across the categories of questions, with MDD and SPC questions presenting more challenges than CTA questions. English responses had lower readability (mean flesch reading ease score 32.7) compared to Chinese responses. Moonshot-v1 provided the highest rate of safety disclaimers (98.7% English, 100% Chinese).

**Conclusion:**

LLMs showed varying degrees of potential for acquiring CM knowledge. The performance of DeepSeek-v3 and DeepSeek-r1 is satisfactory. Optimizing LLMs to become effective tools for disseminating CM information is an important direction for future development.

## 1 Introduction

Chinese medicine (CM), characterized by the theory of yin-yang balance and holistic approaches, is a medical system that evolved from ancient Chinese folk experience and clinical practice. Its therapeutic methods include acupuncture, moxibustion, cupping therapy, gua sha, massage (tuina), and herbal medicine, which are employed for both disease treatment and health maintenance (Ma et al., 2021; Zhang P. et al., 2023; Augustin et al., 2020). To date, CM has established its presence in 196 countries and regions worldwide, serving more than one-third of the global population. According to the World Health Organization (WHO), 113 member states officially recognize and use acupuncture as a distinctive CM therapeutic modality. In addition, China has established specialized CM cooperation agreements with more than 40 countries and regions (Traditional Chinese medicine gains foothold in 196 countries, regions, 2025; Li et al., 2024). However, despite its remarkable global expansion, it continues to face numerous challenges in its transmission and innovation processes. Its complex knowledge system, highly individualized treatment methods, and reliance on personal experience make standardizing and modernizing CM knowledge difficult (O’Brien et al., 2009; Zhang Q. et al., 2021; Tian et al., 2024; Xiong et al., 2024). Large amounts of pertinent information have surfaced online due to the increased public interest in CM, but it can be challenging to estimate the reliability of the information (Zhao et al., 2019; Xiong et al., 2021; Swire-Thompson and Lazer, 2020; Cyranoski, 2018; Scheufele and Krause, 2019). When seeking CM treatment, patients frequently encounter two challenges: it can be difficult to determine the validity of information found online, and to locate high-quality, low-cost CM treatment in the real world (Zhang et al., 2017; Wang et al., 2021).

A growing number of patients are using large language models (LLMs) to get health information (Thirunavukarasu et al., 2023; Laymouna et al., 2024; Ong et al., 2024b). LLMs have great potential in the field of medicine thanks to their ability to digest massive volumes of information fast, respond instantly around-the-clock, and process information cross-lingually and cross-culturally (Shi et al., 2024). Furthermore, LLMs are able to provide users at various levels with tailored responses and succinct, understandable explanations of complicated CM ideas based on their needs (Zhang S. et al., 2023; Song et al., 2024; Zhou E. et al., 2024). However, the very qualities that make LLMs appealing to patients can paradoxically exacerbate the spread of inaccurate medical information. The same capabilities that enable LLMs to communicate complex CM concepts effectively can also cause potentially harmful misinformation to appear credible and authoritative to users lacking the expertise to critically evaluate content. The quality of the output is also directly impacted by the limited knowledge base of LLMs and their reliance on training data (Meskó and Topol, 2023; Thirunavukarasu et al., 2023; Freyer et al., 2024; Ong et al., 2024a). Given this duality, it becomes essential to systematically evaluate the accuracy, readability, and presence of appropriate safety disclaimers in LLM-generated CM information, in order to mitigate potential risks while harnessing the benefits of these powerful tools.

The ability of LLMs to provide reliable information has been investigated in several areas of medicine (Chen D. et al., 2024; Deng et al., 2024; Scaff et al., 2024; Shi et al., 2024; Yalamanchili et al., 2024). However, the performance of LLMs in answering clinical practice guidelines (CPGs) questions in CM needs further investigation. The aim of this study was to systematically assess LLMs’ adherence to CPGs when answering CM-related questions and provide a reference for global users seeking CM-related information through LLMs. Three interdependent evaluation metrics (accuracy, readability, and safety disclaimers) were employed to determine practical applicability in clinical contexts, extending conventional accuracy-focused medical assessments used in licensing examination scenarios (Wang X. et al., 2023; Tseng et al., 2025).

## 2 Materials and methods

**FIGURE 1:**
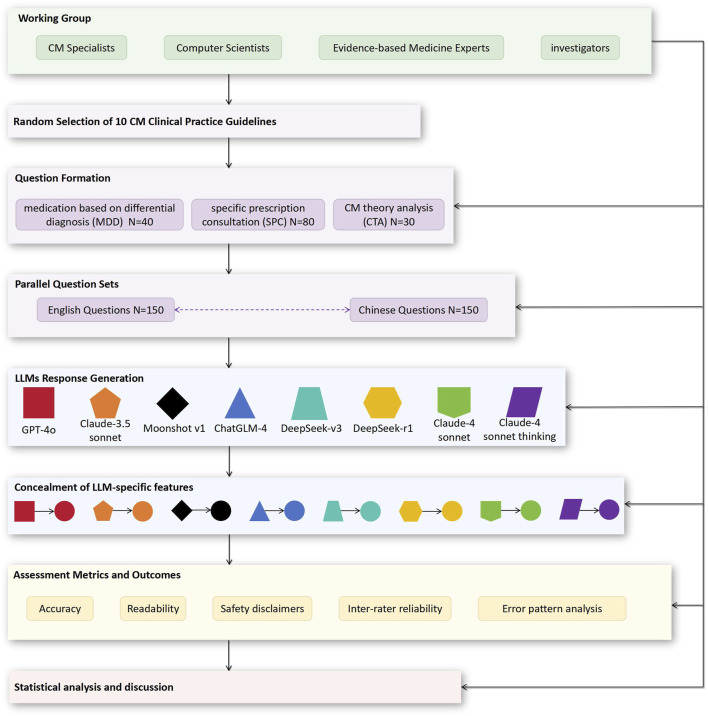
Flow diagram of the study process. CM, Chinese Medicine; LLMs, large language models.

This cross-sectional study adhered to the Strengthening the Reporting of Observational Studies in Epidemiology (STROBE) guidelines for reporting observational studies (von Elm et al., 2007). The institutional review board exempted the study from ethical review as it used only published data. Figure 1 illustrates the study process.

### 2.1 Formation of the working group

A multidisciplinary team comprising five CM specialists, two computer scientists, two experts in evidence-based medicine, and a core group of five investigators carried out this study. The CM experts, assisted by the computer scientists, designed and refined the questions, while the evidence-based medicine experts oversaw the process. The core group collected CPGs, conducted research, and performed statistical analysis under the guidance of the experts.

### 2.2 Data source

The China National Knowledge Infrastructure (CNKI) is the largest academic database in China, aggregating scholarly resources from a variety of fields (The China National Knowledge Infrastructure, 2025). We searched CNKI for CPGs. In this study, CPGs were defined as recommendations based on systematic review of evidence and balanced consideration of benefits and harms of different interventions to provide optimal healthcare services for patients (Clinical Practice Guidelines We Can Trust, 2011). To ensure comprehensive coverage of CM practices, methodological rigor, and reliable results that validly reflect the integrated nature of CM therapeutic modalities, we applied the following criteria: 1) the subject of the CPG is CM, excluding CPGs for Chinese patent medicine (which focus solely on pharmaceutical products without covering the full spectrum of CM therapeutic modalities such as herbal formulations, acupuncture, and traditional exercises) (Wang J.-X. et al., 2024) and integrated Chinese-Western medicine (to maintain focus on CM); 2) the affiliated journals are included in the Peking University List of Chinese Core Journals to ensure publication quality and rigorous peer-review standards; and 3) published from January 2021 to July 2024 to ensure currency of recommendations. All eligible CPGs were assigned sequential numbers. We used Microsoft Excel 2023 to generate random numbers, and selected the CPGs corresponding to the first 10 random numbers as the source of the question sets. For each CPG, three types of questions were constructed based on previous research and expert consensus: medication based on differential diagnosis (MDD) questions focusing on syndrome differentiation scenarios, specific prescription consultation (SPC) questions involving specific clinical applications, and CM theory analysis (CTA) questions examining theoretical principles (Chen C. et al., 2025; Chen Z. et al., 2025; He et al., 2025; Huang et al., 2025). We constructed parallel question sets in English and Chinese with a uniform format (Supplementary Methods and Table S1).

### 2.3 Application of LLMs

Eight LLMs were selected based on audience level and accessibility (GPT-4o, Claude-3.5 Sonnet, Moonshot-v1, ChatGLM-4, DeepSeek-v3, DeepSeek-r1, Claude-4 sonnet, and Claude-4 sonnet thinking; Supplementary Table S2). We evaluated the LLMs’ baseline performance using their pre-existing knowledge, without any additional training or fine-tuning with the CPGs. All questions were entered into the LLMs by a single investigator in as short time as possible. Each question was designed using the zero-shot approach, as this was intended to be a no-frills simulation of how a regular user would ask a question. For MDD questions, we additionally employed prompts requiring LLMs to provide rationale to compare performance with and without justification. To avoid potential interference between responses, each question was entered independently using the new chat feature, and each input/output process was submitted anonymously (without IP identification). Each response was recorded verbatim as text, without LLM features, and compared to the recommendations in the CPGs. Any input that was interrupted due to network issues or operator error was excluded and immediately redone.

### 2.4 Evaluation metrics and outcomes

#### 2.4.1 Accuracy

Responses to LLMs in terms of accuracy were scored according to CPGs on five-point Likert scale, with 1 = completely inaccurate; 2 = more inaccurate than accurate; 3 = approximate balance between accuracy and inaccuracy; 4 = more accurate than inaccurate; 5 = completely accurate. For MDD questions, two CM experts independently scored the questions. Conflicts in the scoring process were resolved by a third expert. For the SPC and CTA questions, five CM experts independently rated the accuracy of LLMs’ responses according to the CPG. The experts were blinded to which LLM generated each response during their evaluation. We calculated the final score for each response using the principle of majority agreement in light of the possible subjective bias. If four or more experts disagreed with the answer, we used the most stringent scoring criteria and assigned the lowest score among the five experts (Deng et al., 2024). The accuracy scoring process was conducted in four rounds, with each round occurring on a different day and a 24-h washout period between rounds.

#### 2.4.2 Readability

Given the potential bias and subjectivity of medical professionals, standardized readability assessment platforms were used to objectively evaluate the readability of the output content of LLMs. For English texts, we used the Flesch Reading Ease Score (FRES), which ranges from 0 to 100, with higher scores indicating better readability (Kincaid et al., 1975). The FRES assessment was performed using an online platform (https://datayze.com/readability-analyzer). For Chinese texts, we used the Chinese Readability Platform (CRP) (Cheng et al., 2020; Shi et al., 2024; The Chinese Readability Platform, 2025). Unlike the FRES, the CRP does not have a fixed scale range but corresponds to educational grade levels, with higher scores indicating lower readability of the text. CRP scores are administered through the official website (Supplementary Table S3). We also counted the response length of the output content of the LLMs.

#### 2.4.3 Safety disclaimers

Safety disclaimers are defined as information that appears in the output content of LLMs that alerts users to potential risks or promotes safe behaviors in specific situations. This included, but was not limited to, recommendations to seek medical attention if symptoms persist or worsen, reminding users that the information provided is not a substitute for professional medical advice, and emphasizing the importance of consulting with a CM practitioner or other healthcare professional before pursuing any treatment options. Two independent researchers coded each LLM’s responses and categorized them as either “with disclaimer (YES)” or “without disclaimer (NO).” A third researcher adjudicated any disagreements.

#### 2.4.4 Inter-rater reliability

The degree of agreement between raters when rating the same item is known as inter-rater reliability. For the MDD questions and safety disclaimers, which were evaluated by two independent raters, we used Cohen’s kappa coefficient to measure agreement. For the SPC and CTA questions, which were evaluated by five expert raters, we used Fleiss’ kappa coefficient to assess multi-rater agreement. Both kappa statistics range from −1 to +1, where one represents perfect agreement, 0 represents agreement by chance, and negative values indicate agreement worse than chance. Kappa values can be interpreted as follows: 0.0–0.20 indicates slight agreement; 0.21–0.40 indicates fair agreement; 0.41–0.60 indicates moderate agreement; 0.61–0.80 indicates substantial agreement; and 0.81–1.00 indicates almost perfect agreement.

#### 2.4.5 Error pattern analysis

To elucidate the performance characteristics and potential limitations of LLMs in answering CM questions, five investigators conducted consensus-based discussions on low-scoring responses, reaching agreement through collective review and discussion to identify common error patterns in how LLMs handled CM questions.

### 2.5 Statistical analysis

This study used Excel 2023 (Microsoft Corp., Redmond, WA, United States), IBM SPSS Statistics for Windows, Version 27.0 (IBM Corp., Armonk, NY, United States), R version 4.3.0 (R Foundation for Statistical Computing, Vienna, Austria), and Origin 2024 (OriginLab Corp., Northampton, MA, United States) for data organization, statistical analysis and data visualization, respectively. Median and interquartile range (IQR) were used for non-normally distributed data, and mean ± standard deviation (SD) were used for normally distributed data. The Kolmogorov-Smirnov test was used to assess the normality of the data distribution. For non-normally distributed data, multiple group comparisons were performed using the Kruskal–Wallis test with Dunn’s *post hoc* test. The Wilcoxon signed-rank test was used to compare score differences between English and Chinese outputs of LLMs. The Benjamini–Hochberg correction method was applied to control the false discovery rate (FDR) within each question type for multiple hypothesis testing (Benjamini and Hochberg, 1995). Statistical significance was set at *p* = 0.05.

## 3 Results

We randomly selected ten CPGs in CM published between January 2021 and July 2024 and fulfilling the inclusion criteria. These CPGs covered a variety of conditions: endometriosis-associated pain (Chen J. et al., 2024), diabetic peripheral vascular disease (Fan et al., 2023), viral pneumonia in children (Yuan et al., 2023), acute myocardial infarction (Zhang M. et al., 2021), allergic rhinitis in children (Wang S. et al., 2023), coronary microvascular disease (Liu et al., 2023), abnormal uterine bleeding (Du et al., 2021), acute tracheobronchitis (Li et al., 2021), dry eye syndrome (Peng et al., 2022), and gout (Wen et al., 2024). Based on the contents of these CPGs, we developed a total of 150 questions: 40 on MDD, 80 on SPC, and 30 on CTA. Each LLM received all 150 questions in both English and Chinese.

### 3.1 Accuracy

**FIGURE 2:**
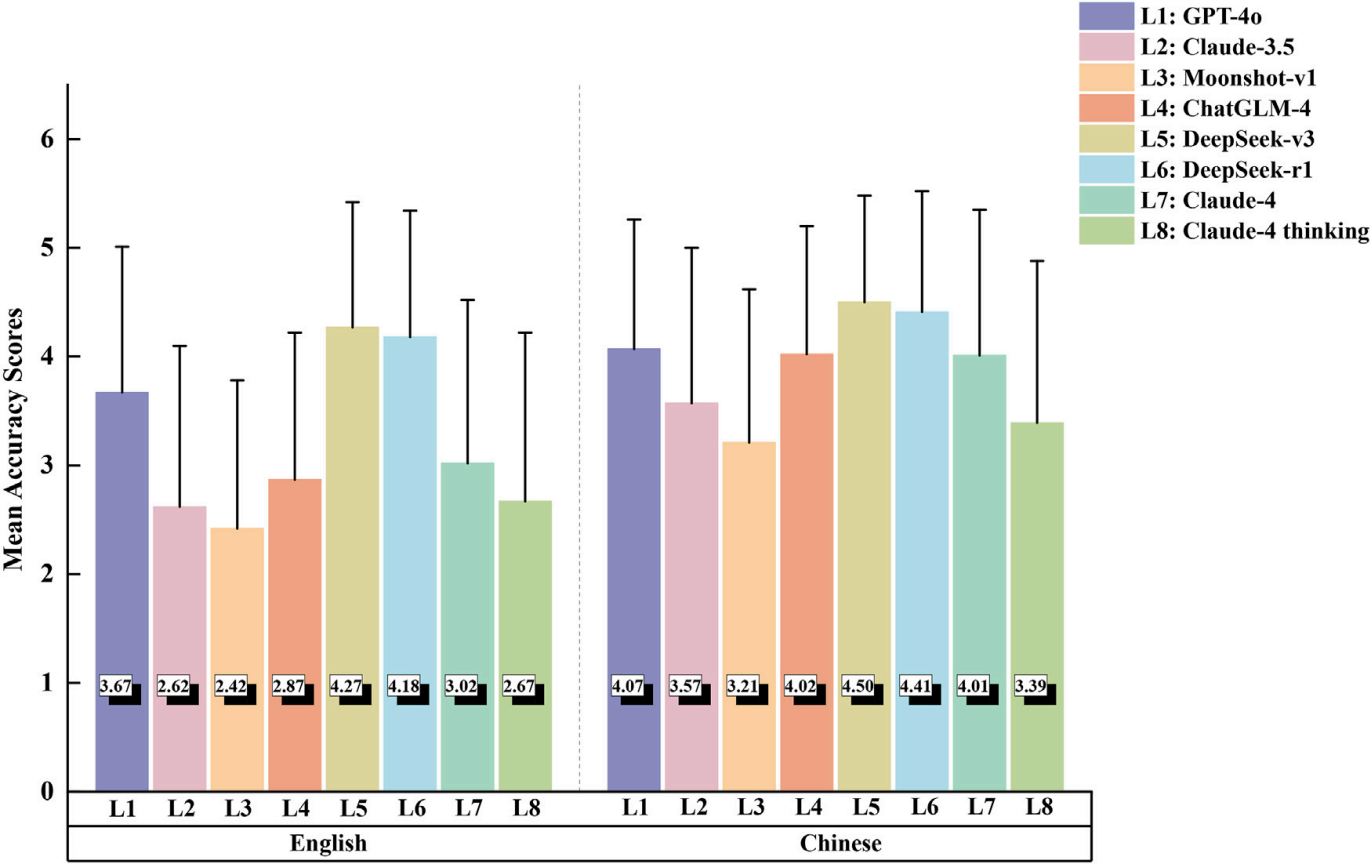
Mean accuracy scores for large language models in English and Chinese responses. Bars represent mean scores, and error bars indicate standard deviation.

Table 1 presents the detailed accuracy scores for the eight LLMs in both English and Chinese language responses, with the mean accuracy scores visualized in Figure 2. The complete results of Dunn’s *post hoc* tests for pairwise comparisons are provided in Supplementary Table S4.

**TABLE 1:** Aaccuracy scores for the eight LLMs.

LLMs	GPT-4o	Claude-3.5	Moonshot-v1	ChatGLM-4	DeepSeek-v3	DeepSeek-r1	Claude-4	Claude-4 thinking	*p*-value
English									
MDD (N = 40)									
Mean (SD)	3.40 (1.93)	2.95 (1.78)	2.20 (1.68)	2.20 (1.74)	3.95 (1.57)	4.10 (1.57)	3.15 (1.59)	2.95 (1.78)	<0.001
Median (IQR)	5.00 (1.00–5.00)	3.00 (1.00–5.00)	1.00 (1.00–3.00)	1.00 (1.00–4.50)	5.00 (3.00–5.00)	5.00 (3.00–5.00)	3.00 (1.00–5.00)	3.00 (1.00–5.00)	
SPC (N = 80)									
Mean (SD)	3.94 (0.94)	2.14 (1.27)	2.41 (1.22)	3.27 (1.05)	4.52 (1.85)	4.20 (0.10)	2.63 (1.39)	2.14 (1.24)	<0.001
Median (IQR)	4.30 (1.00–5.00)	1.70 (1.00–3.55)	2.20 (1.30–3.30)	3.30 (2.30–4.00)	5.00 (4.33–5.00)	4.40 (3.80–5.00)	3.05 (1.00–3.80)	1.70 (1.00–3.63)	
CTA (N = 30)									
Mean (SD)	3.30 (1.18)	3.27 (1.29)	2.73 (1.23)	2.70 (1.15)	4.03 (1.07)	4.23 (0.94)	3.90 (1.27)	3.73 (1.31)	<0.001
Median (IQR)	3.00 (3.00–4.00)	3.00 (2.00–4.00)	3.00 (1.75–3.00)	3.00 (2.00–3.00)	4.00 (3.00–5.00)	5.00 (3.00–5.00)	4.00 (3.00–5.00)	4.00 (3.00–5.00)	
Total (N = 150)									
Mean (SD)	3.67 (1.34)	2.62 (1.49)	2.42 (1.36)	2.87 (1.36)	4.27 (1.15)	4.18 (1.16)	3.02 (1.50)	2.67 (1.55)	<0.001
Median (IQR)	4.00 (2.98–5.00)	2.70 (1.00–3.80)	2.20 (1.00–3.03)	3.00 (1.60–4.00)	5.00 (4.00–5.00)	5.00 (3.70–5.00)	3.00 (1.00–4.16)	2.85 (1.00–3.80)	
Chinese									
MDD (N = 40)									
Mean (SD)	3.90 (1.81)	3.40 (1.93)	3.15 (1.99)	4.05 (1.69)	4.10 (1.43)	4.15 (1.49)	3.75 (1.55)	3.50 (1.49)	0.070
Median (IQR)	5.00 (1.00–5.00)	5.00 (1.00–5.00)	5.00 (1.00–5.00)	5.00 (3.50–5.00)	5.00 (3.00–5.00)	5.00 (3.00–5.00)	5.00 (3.00–5.00)	3.00 (3.00–5.00)	
SPC (N = 80)									
Mean (SD)	4.25 (0.79)	3.66 (1.27)	3.07 (1.17)	4.09 (0.88)	4.62 (0.75)	4.43 (1.01)	3.96 (1.33)	2.90 (1.47)	<0.001
Median (IQR)	4.30 (3.60–5.00)	4.30 (2.60–5.00)	3.05 (2.20–4.10)	4.30 (3.60–5.00)	5.00 (4.70–5.00)	5.00 (4.30–5.00)	4.55 (3.33–5.00)	3.10 (1.30–4.10)	
CTA (N = 30)									
Mean (SD)	3.80 (1.06)	3.57 (1.07)	3.67 (0.96)	3.77 (1.07)	4.70 (0.57)	4.70 (0.36)	4.50 (0.86)	4.53 (0.73)	<0.001
Median (IQR)	4.00 (3.00–5.00)	3.00 (3.00–5.00)	3.00 (3.00–5.00)	4.00 (3.00–5.00)	5.00 (4.75–5.00)	5.00 (4.75–5.00)	5.00 (4.00–5.00)	5.00 (4.00–5.00)	
Total (N = 150)									
Mean (SD)	4.07 (1.20)	3.57 (1.44)	3.21 (1.42)	4.02 (1.19)	4.50 (0.98)	4.41 (1.11)	4.01 (1.34)	3.39 (1.49)	<0.001
Median (IQR)	4.30 (3.60–5.00)	4.15 (2.40–5.00)	3.05 (1.98–4.70)	4.30 (3.10–5.00)	5.00 (4.30–5.00)	5.00 (4.30–5.00)	5.00 (3.00–5.00)	3.80 (2.28–5.00)	

N, number of questions; LLMs, large language models; IQR, interquartile range; SD, standard deviation; K-W H, Kruskal–Wallis H test statistic; CM, chinese medicine; MDD, medication based on differential diagnosis questions; SPC, specific prescription consultation questions; CTA, CM, theory analysis questions.

The Kruskal–Wallis test revealed significant differences in performance among the eight large language models for English responses (H = 241.46, *p* < 0.001). DeepSeek-v3 and DeepSeek-r1 emerged as the to*p* performers, showing statistically equivalent accuracy (*p* = 0.577) while significantly outperforming all other models (all *p* < 0.01). GPT-4o ranked third among the remaining models, followed by Claude-4 and ChatGLM-4, which demonstrated comparable performance (*p* = 0.282). The lowest accuracy scores were observed in Claude-4 thinking mode, Claude-3.5, and Moonshot-v1, with no significant differences among these three models. Similar patterns emerged in Chinese-language responses, where significant inter-model differences were also detected (Kruskal–Wallis H = 135.49, *p* < 0.001). DeepSeek-v3 and DeepSeek-r1 maintained their superior performance with equivalent accuracy scores (*p* = 0.624), significantly exceeding other LLMs (all *p* < 0.01). Claude-4 demonstrated intermediate performance, showing no significant differences from GPT-4o (*p* = 0.747) or ChatGLM-4 (*p* = 0.540). Moonshot-v1 and Claude-4 thinking mode again exhibited the poorest performance (*p* = 0.217). Notably, all eight LLMs achieved significantly higher accuracy in Chinese compared to English responses (all *p* < 0.05, see Supplementary Table S5).

**FIGURE 3:**
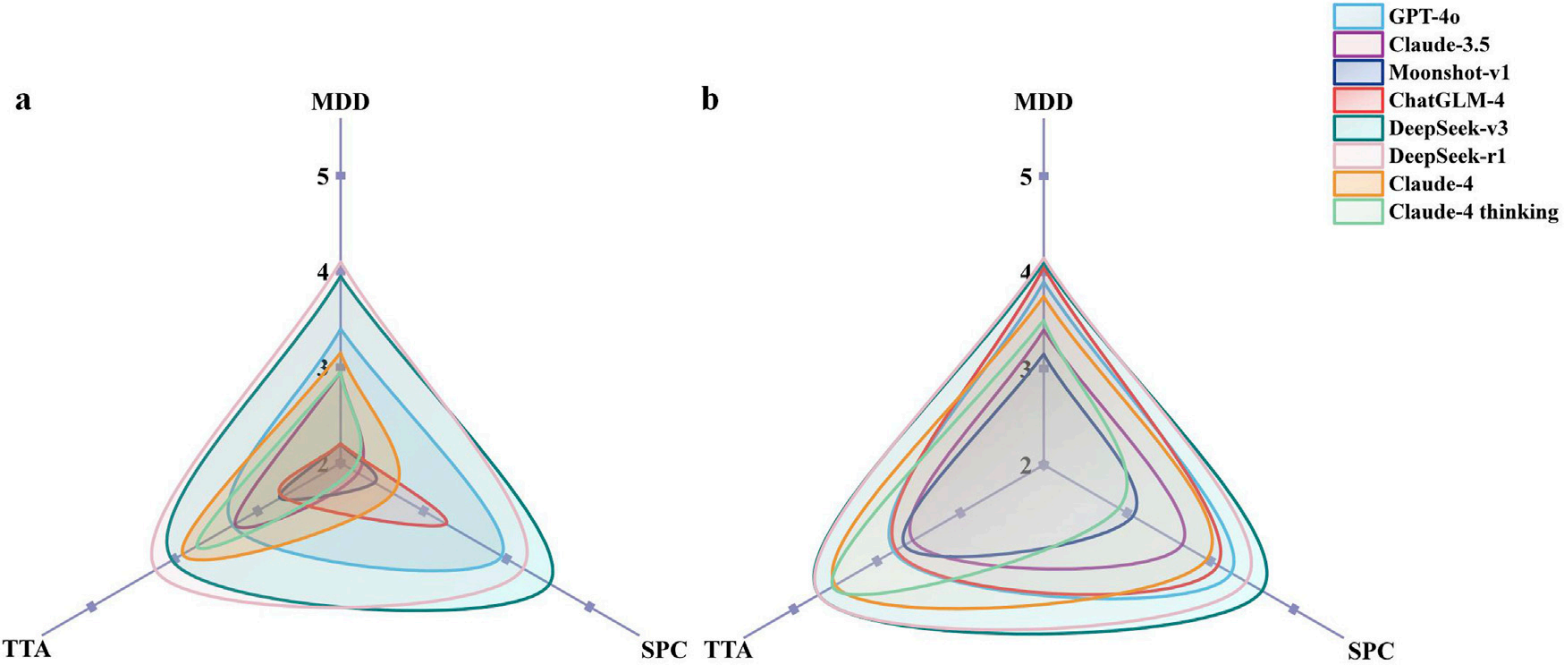
Comparison of the scores for the different categories of questions in English **(a)** and Chinese **(b)**. MDD, Medication based on Differential Diagnosis; SPC, Specific Prescription Consultation; CTA, CM Theory Analysis. Each axis represents the mean accuracy score for a specific question category. The area covered by each LLM’s polygon indicates its overall performance across categories.

Performance varied by question type (Figure 3) In English MDD questions, DeepSeek-r1 and DeepSeek-v3 maintained their leading positions with equivalent performance (*p* = 0.753), both significantly outperforming Moonshot-v1, ChatGLM-4 (all *p* < 0.001), Claude-3.5 (*p* = 0.014 and *p* = 0.036, respectively), and Claude-4 thinking mode (all *p* < 0.001). GPT-4o also demonstrated strong performance, significantly exceeding Moonshot-v1 and ChatGLM-4 (both *p* = 0.014). In contrast, no significant differences were found among the models for Chinese MDD questions (*p* = 0.070). The SPC question analysis revealed DeepSeek-v3 as the standout performer, significantly outperforming all other models except DeepSeek-r1 (*p* = 0.107). DeepSeek-r1 and GPT-4o performed similarly, with no significant difference between them (*p* = 0.126), both significantly outperforming Claude-3.5, Moonshot-v1, ChatGLM-4, and both Claude-4 variants (all *p* < 0.05). Claude-3.5, Moonshot-v1, and Claude-4 thinking mode clustered at the lower end of performance. In Chinese SPC questions, DeepSeek-v3 and DeepSeek-r1 performed best with no significant difference between them (*p* = 0.364). Meanwhile, GPT-4o, ChatGLM-4, and Claude-4 demonstrated comparable intermediate performance. Lower-performing models in SPC questions frequently prioritized liability disclaimers over specific treatment recommendations. For CTA questions in English, DeepSeek-r1 achieved the highest performance, significantly outperforming most other models except for DeepSeek-v3 (*p* = 0.649). In Chinese, DeepSeek-v3 and DeepSeek-r1 demonstrated equally superior performance (*p* = 0.999) and significantly outperformed GPT-4o, Claude-3.5, Moonshot-v1, and ChatGLM-4 (all *p* < 0.001). Claude-4 and Claude-4 Thinking demonstrated strong performance, performing significantly better than earlier generation models, though comparably to the DeepSeek models (all *p* > 0.05).

We further investigated the impact of requiring a rationale on LLM performance on MDD questions. Analyzing the combined performance of all models across English and Chinese responses revealed that providing justification significantly improved accuracy scores (*p* = 0.019, see Supplementary Table S6).

### 3.2 Readability

Overall, the readability of the English responses had a mean FRES of 32.7, which is rated as “difficult” and equivalent to requiring at least the reading level of a U.S. college student (Supplementary Table S7). DeepSeek-v3 achieved the highest readability score (40.10 ± 13.27), while Claude-4 thinking showed the lowest readability (20.66 ± 14.59). The other LLMs’ readability scores were GPT-4o (39.33 ± 12.71), DeepSeek-r1 (36.43 ± 10.63), Moonshot-v1 (36.21 ± 9.03), ChatGLM-4 (33.63 ± 13.47), Claude-3.5 (30.52 ± 14.36), and Claude-4 (24.53 ± 16.28). In contrast, the Chinese responses were easier to understand, with an overall mean readability score of 11.74, comparable to junior high school level in China. At the average level, the readability differences among models were relatively small, with scores ranging from 10.99 (ChatGLM-4) to 12.70 (DeepSeek-r1).

### 3.3 Safety disclaimers

**FIGURE 4:**
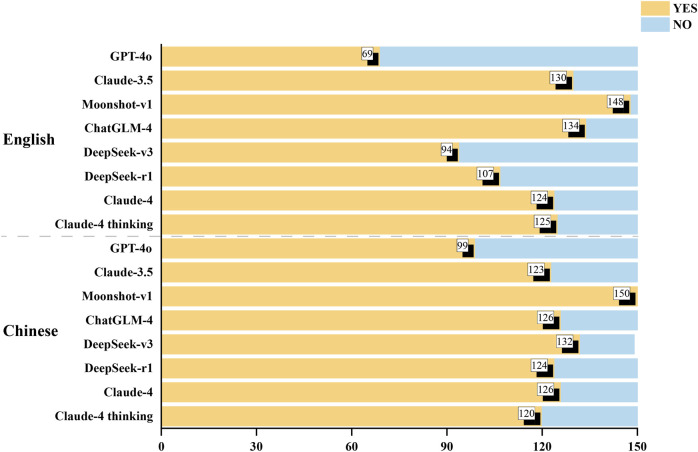
Number of safety disclaimers included in the LLMs’ responses. LLMs, large language models.

The presence of safety disclaimers in the responses generated by the 8 LLMs is shown in Figure 4. Moonshot-v1 performed significantly better than the other seven LLMs in providing safety-related information, with 98.7% of the English outputs and 100% of the Chinese outputs containing safety disclaimer, followed by ChatGLM-4 (89.3% English, 84.0% Chinese), Claude-3.5 (86.7% English, 82.0% Chinese), Claude-4 (82.7% English, 84.0% Chinese), Claude-4 thinking mode (83.3% English, 80.0% Chinese), DeepSeek-r1 (71.3% English, 82.7% Chinese), DeepSeek-v3 (62.7% English, 88.0% Chinese), and GPT-4o (46.0% English, 66.0% Chinese).

### 3.4 Inter-rater reliability

We assessed inter-rater agreement using kappa values. The two experts were in perfect agreement with each other regarding the MDD questions. For the SPC and CTA questions, Fleiss’ kappa analysis showed a significantly high level of agreement between the five raters (*κ* = 0.698, 95% CI: 0.690–0.706). For the safety disclaimers, Cohen’s kappa analysis showed almost perfect agreement between the two investigators (*κ* = 0.801, 95% CI: 0.775–0.852).

### 3.5 Error pattern analysis

The error pattern analysis identified several common patterns that led to reduced scores. Two prominent issues emerged: first, the LLMs tended to provide generic responses without addressing patients’ specific symptoms and conditions; second, some responses focused excessively on liability disclaimers at the expense of substantive medical information (Supplementary Table S8).

## 4 Discussion

The scope of applications for LLMs in multiple medical fields is continuously expanding. Related research has evaluated LLMs’ performance in answering questions related to Western medical CPGs, medical licensing exams, and diagnostic reasoning, demonstrating promising application prospects (Beam et al., 2023; Ho et al., 2024; Longwell et al., 2024; Zhou S. et al., 2024). Given that CM has spread to 196 countries and regions worldwide, with more than one-third of the global population receiving CM treatment, its global influence continues to expand (The State Council of the People’s Republic of China, 2024). However, the exploration of specifically applying LLMs to CPGs in CM remains relatively limited. In the era of rapid artificial intelligence (AI) development, exploring the potential applications of LLMs in CM knowledge acquisition and dissemination has significant practical implications. In this cross-sectional study, we evaluated the performance of eight cutting-edge LLMs in answering questions related to CM based on CPGs.

Our results demonstrate that LLMs have great potential in CM knowledge acquisition, though with notable performance variations across models. DeepSeek-v3 and DeepSeek-r1 consistently emerged as the top performers across all question types and languages, significantly outperforming other LLMs. This superior performance of DeepSeek models may reflect their advanced reasoning capabilities and potentially better training on CM content, although the specific contributing factors warrant further investigation. Surprisingly, our study revealed minimal performance differences between reasoning models and their standard counterparts, despite the theoretical advantages of enhanced reasoning capabilities. This unexpected finding may be attributed to the nature of CM knowledge assessment, which relies primarily on factual recall and pattern recognition rather than complex multi-step reasoning. Additionally, the relatively straightforward question formats in our evaluation may not have fully leveraged the advanced reasoning capabilities that distinguish these specialized models.

The performance of the remaining models varied by language. GPT-4o demonstrated strong intermediate performance in English responses, followed by Claude-4 and ChatGLM-4, which showed comparable results. In Chinese responses, Claude-4 performed at an intermediate level, showing no significant differences compared to GPT-4o or ChatGLM-4. Notably, ChatGLM-4, a model optimized for the Chinese language, performed similarly to internationally developed models, such as GPT-4o and Claude-4, in both languages. This suggests that, while language-specific optimization offers some advantages, it may not be the primary factor in determining performance in specialized medical domains. The consistently lower performance of Claude-3.5 and Moonshot-v1 compared to newer generation models highlights the rapid advancement in LLM capabilities. Extrapolating from these results, model performance in Chinese medicine applications will likely continue to improve through larger training datasets and technical innovations.

The superior performance of all eight LLMs in Chinese, compared to English, supports our hypothesis that CM concepts are more accurately conveyed in their original linguistic context. This difference likely reflects the fact that CM concepts were originally developed and defined in Chinese. Consequently, Chinese-language responses can convey these concepts more accurately without the potential loss of meaning that can occur during translation to English.

Comparisons across different question types reveal that large language models face varying degrees of challenges when processing CM knowledge of different levels of complexity. For MMD and SPC questions in particular, although some models perform relatively well (e.g., DeepSeek-v3 and DeepSeek-r1), we believe that providing specific treatment recommendations for patients remains a major challenge for large language models. Our error pattern analysis shows that poorly performing models (e.g., Claude-3.5, Moonshot-v1, and Claude-4 thinking) tend to provide disclaimers rather than specific treatment recommendations, often stating that they are not qualified to provide professional medical advice and suggesting users consult medical professionals. This phenomenon may result from the increasing emphasis on medical ethics and safety considerations in the underlying design of large language models, or it could be an active avoidance strategy caused by model performance limitations. While this approach reduces medical risks to some extent, it also limits the practical utility of models in professional medical scenarios, especially in situations requiring specific clinical guidance. In contrast, our analysis of CTA questions reveals that LLMs perform comparatively well in processing theoretical knowledge. This stark difference from the aforementioned question types further underscores the challenge of translating theoretical knowledge into practical clinical recommendations.

Additionally, although our zero-shot prompting approach realistically mimics how typical users interact with LLMs, it is important to note that this method may underestimate the models’ potential, which could be realized through more sophisticated prompting strategies or model customization (Wang L. et al., 2024). Our study found that simply asking LLMs to explain their answers, a basic prompt optimization, significantly improved overall accuracy. It remains unclear whether structured prompt engineering through few-shot learning, chain-of-thought reasoning, or fine-tuning could significantly improve the reliability of LLMs as supplementary medical information tools.

Regarding readability, in contrast to the Chinese responses, which were easily readable, the readability of the English responses was rated as “difficult,” which is equivalent to require at least the reading level of a U.S. college student. This finding is consistent with that of Yalamanchili et al. (2024), suggesting that health-related information generated by LLMs may be beyond the comprehension of the general public, although this problem can be ameliorated by secondary input. In terms of safety disclaimers, we found that nearly all responses generated by the Moonshot-v1 contain safety disclaimers, a proportion significantly higher than other models. Since these responses may significantly influence patients’ behavior and their likelihood of seeking professional medical help, it is crucial to emphasize the importance of such disclaimers (Yu et al., 2025). However, while these disclaimers serve a critical protective role by encouraging users to seek professional medical advice, it remains unknown whether overly frequent disclaimers might affect the informativeness and practicality of the responses. Future research should explore the balance between safety measures and practical utility, including whether users may develop a “desensitization” response to repetitive disclaimers, and how to optimize disclaimer strategies to enhance user experience while ensuring safety.

Integrating LLMs into CM practice raises important questions about practitioners’ evolving role in the AI era (Bhayana et al., 2024; McCoy et al., 2024). Our findings suggest that LLMs could serve as valuable supplementary tools rather than replacements, functioning as intelligent knowledge repositories and educational aids, particularly given their superior performance in processing theoretical knowledge. However, the challenges LLMs face with specific treatment planning underscore that individualized clinical decision-making, central to CM philosophy, requires human judgment that integrating patient-specific factors and clinical experience that current artificial intelligence cannot fully replicate. As AI tools become prevalent, CM practitioners will need to develop new competencies including AI literacy, critical evaluation skills for AI-generated recommendations, and enhanced communication abilities to explain AI-assisted decisions while maintaining therapeutic relationships. The future of CM practice likely lies in a collaborative model in which practitioners leverage AI capabilities while maintaining their essential roles as clinical decision-makers, patient advocates, and guardians of CM’s holistic treatment philosophy.

To our knowledge, this is the first study to systematically evaluate the performance of LLMs in answering questions about CPGs for CM in both English and Chinese context. Our results tentatively suggests that LLMs may offer an effective way to acquire knowledge about CM under the premise of ensuring safety. However, this study has also several limitations. First, the evaluation based on CPGs may not fully capture the comprehensive and individualized nature of CM clinical practice. Second, the sample of 150 questions may not be fully representative of the breadth and complexity of CM knowledge. Third, the evaluation process may be to some extent subjective despite the use of the two-by-two independent and majority consensus methods. Fourth, the readability assessment tools used in this study have inherent limitations. General readability metrics like FRES and CRP may not fully capture the domain-specific complexity of CM concepts, cultural nuances, and specialized terminology that are integral to CM. Finally, the rapid updating of the LLMs may affect the timeliness of study results, calling for cautious interpretation of results.

## 5 Conclusion

LLMs demonstrate varying degrees of potential as tools for acquiring CM related knowledge, with DeepSeek-v3 and DeepSeek-r1 performing best in both English and Chinese contexts. All models demonstrated superior performance in Chinese responses compared to English. LLMs excel at processing CM theoretical knowledge but face challenges in providing specific treatment recommendations. LLMs can serve as supplementary tools for CM knowledge acquisition, but must be used with appropriate safety considerations. Future research should focus on optimizing LLM application strategies in the field of CM.

## Data Availability

The original contributions presented in the study are included in the article/Supplementary Material, further inquiries can be directed to the corresponding author.
