# Supplementary material for: Assessing the adherence of large language models to clinical practice guidelines in Chinese medicine: a content analysis
Source: Front Pharmacol. 2025 Jul 25;16:1649041. doi: 10.3389/fphar.2025.1649041 (PMC12331602; doi:10.3389/fphar.2025.1649041)
Supplement: Supplementary file 1 [file Supplementaryfile1.pdf]

Assessing the Adherence of Large Language Models to Clinical Practice Guidelines in Chinese Medicine: A Content Analysis

Contents

eMethods 1

Supplementary Table S1. Types of questions 3

Supplementary Table S2. Large language models used in this study 4

Supplementary Table S3. Readability Tools 5

Supplementary Table S4. Results of Dunn's post hoc tests for pairwise comparisons of accuracy scores among the eight LLMs 6

Supplementary Table S5. Comparison of accuracy scores between English and Chinese responses for each LLM 13

Supplementary Table S6. Comparison of 8 LLMs Performance on MDD Questions With and Without Rationale Requirements 14

Supplementary Table S7. Readability of the responses of the LLMs 15

Supplementary Table S8. Examples of reasons contributing to score deduction in llm outputs 16

eMethods

Question Construction Process

Framework Development and Consensus Building. To establish preliminary consensus, a meeting was organized with participants including three Chinese medicine (CM) experts, two methodology experts, and multiple investigators. During this foundational meeting, the team established conceptual frameworks for three categories of questions: medication based on differential diagnosis (MDD) questions, focusing on syndrome differentiation scenarios (originally syndrome differentiation and medication (SDM) questions, modified per reviewer #1's suggestion); specific prescription consultation (SPC) questions, involving specific clinical applications; and CM theory analysis (CTA) questions, examining theoretical principles. The meeting collectively established definitions, standardized templates, format guidelines, quality standards, and general workflow procedures for each question type.

Content Extraction and Categorization. Two independent investigators conducted comprehensive reviews of each clinical practice guideline that had been screened and randomly selected. The review process focused on identifying key clinical scenarios, diagnostic criteria, treatment recommendations, and theoretical foundations. The extracted content was systematically categorized into three different types of questions based on their clinical applications: syndrome differentiation scenarios in MDD questions, specific clinical scenarios in SPC questions, and theoretical principles in CTA questions.

Question Development. Question construction followed standardized templates customized for each category. MDD questions present clinical symptoms and signs, requiring large language models to identify corresponding syndrome patterns and recommend appropriate CM treatment plan. SPC questions describe specific patient scenarios that require prescription adjustments, dosage modifications, or explanations of treatment rationale. CTA questions focus on theoretical interpretations of treatment principles, Chinese herbal properties, or syndrome differentiation logic. Each question includes three core components: comprehensive clinical background or scenario description, specific and focused questions, and expected answer frameworks based on clinical practice guideline recommendations.

Expert Validation and Consensus. Three CM experts independently reviewed all of the constructed CM questions. The validation process ensured the questions were clinically relevant and authentic, consistent with clinical practice guideline recommendations, of an appropriate difficulty level for professional assessment, and clear and precise in formulation. Questions that received unanimous approval from all three experts were retained. Questions on which the experts

disagreed were revised or deleted through written recommendations and structured discussions until unanimous consensus was achieved.

Translation and Question Construction Completion. Two investigators created bilingual Chinese-English versions using DeepL software and GPT-4o-mini. Bilingual CM experts conducted a final review to ensure that the integrity of the CM concepts and clinical scenarios was maintained in both language versions. The final parallel Chinese-English question set was formed through iterative revisions.

Supplementary Table S1. Types of questions

Type	Description	Example	Quantity
MDD	MDD questions focuses on the core practice of CM treatment based on syndrome differentiation, and LLMs must consider the characteristics of the syndrome, the properties of the drugs, and their indications to make rational medication recommendations.	For the pain associated with endometriosis due to Qi stagnation and blood stasis, what Chinese medicine (CM) prescriptions should be used for treatment? Could you recommend several commonly used CM prescriptions, and just tell me the names of the medicines?	40
SPC	SPC questions assess the ability of LLMs to apply herbal prescribing in a specific evidence pattern. This includes rationalization of prescription choices, guidance on specific dosages, and prescription adjustments based on changes in symptoms. Such questions require LLMs to possess not only extensive knowledge of CM, but also clinician-like reasoning skills and the ability to flexibly adjust treatment plans according to specific situations.	For the treatment of endometriosis pain with the pattern of cold coagulation and blood stasis, do you recommend using the Chinese medicine Shaofu Zhuyu Decoction to treat this condition? What are the specific ingredients of this herbal formula? Please answer whether you recommend it and respond to my questions.	80
CTA	The CTA questions are designed to assess the LLMs' depth of understanding and ability to apply the theoretical system of CM. It covers the core knowledge areas of basic CM theories, etiology and pathogenesis, and identification and typing. It requires the categorization of diseases into CM categories, the elucidation of their pathogenesis, or the identification and typing of diseases based on symptomatic manifestations.	The pain associated with endometriosis falls under what category in Chinese Medicine?	30

LLMs, large language models; CM, Chinese Medicine; MDD, Medication based on Differential Diagnosis questions; SPC, Specific Prescription Consultation questions; CTA, CM Theory Analysis questions.

Supplementary Table S2. Large language models used in this study

LLMs	Version	Country	Company	Release Date
GPT-4o	Web	USA	OpenAI	May 2024
Claude-3.5 sonnet	Web	USA	Anthropic	June 2024
Moonshot-v1	Web	China	Moonshot AI	October 2023
ChatGLM-4	Web	China	Zhipu AI	January 2024
DeepSeek-v3	Web	China	DeepSeek	December 2024
DeepSeek-r1	Web	China	DeepSeek	January 2025
Claude-4 sonnet	Web	USA	Anthropic	May 2025
Claude-4 sonnet thinking	Web	USA	Anthropic	May 2025

LLMs, large language models.

Supplementary Table S3. Readability Tools

Evaluation tools of Readability	Historical use	Features	Formula	Website
The Flesch Reading Ease Score (FRES)	An English readability tool developed in the late 1940s to assess a wide range of written materials.	Scores range from 0 to 100. The higher the score, the easier the text is to understand. Low scores indicate that the text is complex to understand. Score ranges: 90-100 (very easy, 11-year-old level), 80-90 (easy, conversational English), 70-80 (fairly easy), 60-70 (13-15 year-old level), 50-60 (fairly difficult), 30-50 (difficult, college level), 0-30 (very difficult, university level).	206.835 - 1.015 × (total words ÷ total sentences) - 84.6 × (total syllables ÷ total words)	https://datayze.com/readability-analyzer
The Chinese Readability Platform (CRP)	A Chinese readability tool based on a corpus of language learning materials to be released in 2020	The higher the CRP value, the less readable the text.	38.36 - 45.65 × Average Word Frequency + 54.92 × Conjunction Ratio - 8.96 × Physical Word Meaning Ratio + 11.13 × Word Meaning Richness - 12.34 × Action Word Meaning Ratio + 0.012 × Sentence Length Variation + 20 × Related Word Meaning Ratio	http://120.27.70.114:8000/analysis_a

Supplementary Table S4. Results of Dunn's post hoc tests for pairwise comparisons of accuracy scores among the eight LLMs

Language	Question Type	N	LLM	Aaccuracy Median (IQR) / Mean (SD)	K-W H(p-value)	Dunn's post hoc test*
English	MDD	40	GPT-4o	5.00 (1.00-5.00) / 3.40 (1.93)	42.20 ($p < 0.001$)	GPT-4o vs Claude-3.5: $p = 0.319$
			Claude-3.5	3.00 (1.00-5.00) / 2.95 (1.78)		**GPT-4o vs Moonshot-v1: $p = 0.014$**
			Moonshot-v1	1.00 (1.00-3.00) / 2.20 (1.68)		**GPT-4o vs ChatGLM-4: $p = 0.014$**
			ChatGLM-4	1.00 (1.00-4.50) / 2.20 (1.74)		GPT-4o vs DeepSeek-v3: $p = 0.260$
			DeepSeek-v3	5.00 (3.00-5.00) / 3.95 (1.57)		GPT-4o vs DeepSeek-r1: $p = 0.128$
			DeepSeek-r1	5.00 (3.00-5.00) / 4.10 (1.57)		GPT-4o vs Claude-4: $p = 0.595$
			Claude-4	3.00 (1.00-5.00) / 3.15 (1.59)		GPT-4o vs Claude-4 thinking: $p = 0.319$
			Claude-4 thinking	3.00 (1.00-5.00) / 2.95 (1.78)		Claude-3.5 vs Moonshot-v1: $p = 0.107$
						Claude-3.5 vs ChatGLM-4: $p = 0.107$
						**Claude-3.5 vs DeepSeek-v3: $p = 0.036$**
						**Claude-3.5 vs DeepSeek-r1: $p = 0.014$**
						Claude-3.5 vs Claude-4: $p = 0.726$
						Claude-3.5 vs Claude-4 thinking: $p = 0.999$
						Moonshot-v1 vs ChatGLM-4: $p = 0.999$
						**Moonshot-v1 vs DeepSeek-v3: $p < 0.001$**
						**Moonshot-v1 vs DeepSeek-r1: $p < 0.001$**
						Moonshot-v1 vs Claude-4: $p = 0.051$
						Moonshot-v1 vs Claude-4 thinking: $p = 0.107$
						**ChatGLM-4 vs DeepSeek-v3: $p < 0.001$**
						**ChatGLM-4 vs DeepSeek-r1: $p < 0.001$**
						ChatGLM-4 vs Claude-4: $p = 0.052$
						ChatGLM-4 vs Claude-4 thinking: $p = 0.107$
						DeepSeek-v3 vs DeepSeek-r1: $p = 0.753$
						DeepSeek-v3 vs Claude-4: $p = 0.088$
						**DeepSeek-v3 vs Claude-4 thinking: $p = 0.036$**
						**DeepSeek-r1 vs Claude-4: $p = 0.041$**
						**DeepSeek-r1 vs Claude-4 thinking: $p = 0.014$**

					Claude-4 vs Claude-4 thinking: $p = 0.726$
SPC	80	GPT-4o	4.30 (1.00-5.00) / 3.94 (0.94)	251.04 ($p < 0.001$)	**GPT-4o vs Claude-3.5: $p < 0.001$**
		Claude-3.5	1.70 (1.00-3.55) / 2.14 (1.27)		**GPT-4o vs Moonshot-v1: $p < 0.001$**
		Moonshot-v1	2.20 (1.30-3.30) / 2.41 (1.22)		**GPT-4o vs ChatGLM-4: $p = 0.004$**
		ChatGLM-4	3.30 (2.30-4.00) / 3.27 (1.05)		**GPT-4o vs DeepSeek-v3: $p = 0.002$**
		DeepSeek-v3	5.00 (4.33-5.00) / 4.52 (1.85)		GPT-4o vs DeepSeek-r1: $p = 0.126$
		DeepSeek-r1	4.40 (3.80-5.00) / 4.20 (0.10)		**GPT-4o vs Claude-4: $p < 0.001$**
		Claude-4	3.05 (1.00-3.80) / 2.63 (1.39)		**GPT-4o vs Claude-4 thinking: $p < 0.001$**
		Claude-4 thinking	1.70 (1.00-3.63) / 2.14 (1.24)		Claude-3.5 vs Moonshot-v1: $p = 0.440$
					**Claude-3.5 vs ChatGLM-4: $p < 0.001$**
					**Claude-3.5 vs DeepSeek-v3: $p < 0.001$**
					**Claude-3.5 vs DeepSeek-r1: $p < 0.001$**
					Claude-3.5 vs Claude-4: $p = 0.107$
					Claude-3.5 vs Claude-4 thinking: $p = 0.778$
					**Moonshot-v1 vs ChatGLM-4: $p = 0.002$**
					**Moonshot-v1 vs DeepSeek-v3: $p < 0.001$**
					**Moonshot-v1 vs DeepSeek-r1: $p < 0.001$**
					Moonshot-v1 vs Claude-4: $p = 0.383$
					Moonshot-v1 vs Claude-4 thinking: $p = 0.312$
					**ChatGLM-4 vs DeepSeek-v3: $p < 0.001$**
					**ChatGLM-4 vs DeepSeek-r1: $p < 0.001$**
					**ChatGLM-4 vs Claude-4: $p = 0.015$**
					**ChatGLM-4 vs Claude-4 thinking: $p < 0.001$**
					DeepSeek-v3 vs DeepSeek-r1: $p = 0.107$
					**DeepSeek-v3 vs Claude-4: $p < 0.001$**
					**DeepSeek-v3 vs Claude-4 thinking: $p < 0.001$**
					**DeepSeek-r1 vs Claude-4: $p < 0.001$**
					**DeepSeek-r1 vs Claude-4 thinking: $p < 0.001$**
					Claude-4 vs Claude-4 thinking: $p = 0.060$
CTA	30	GPT-4o	3.00 (3.00-4.00) / 3.30 (1.18)	43.34 ($p < 0.001$)	GPT-4o vs Claude-3.5: $p = 0.896$

		Claude-3.5	3.00 (2.00-4.00) / 3.27 (1.29)		GPT-4o vs Moonshot-v1: $p = 0.188$
		Moonshot-v1	3.00 (1.75-3.00) / 2.73 (1.23		GPT-4o vs ChatGLM-4: $p = 0.151$
		ChatGLM-4	3.00 (2.00-3.00) / 2.70 (1.15)		GPT-4o vs DeepSeek-v3: $p = 0.051$
		DeepSeek-v3	4.00 (3.00-5.00) / 4.03 (1.07)		**GPT-4o vs DeepSeek-r1: $p = 0.011$**
		DeepSeek-r1	5.00 (3.00-5.00) / 4.23 (0.94)		GPT-4o vs Claude-4: $p = 0.106$
		Claude-4	4.00 (3.00-5.00) / 3.90 (1.27)		GPT-4o vs Claude-4 thinking: $p = 0.215$
		Claude-4 thinking	4.00 (3.00-5.00) / 3.73 (1.31)		Claude-3.5 vs Moonshot-v1: $p = 0.206$
					Claude-3.5 vs ChatGLM-4: $p = 0.187$
					**Claude-3.5 vs DeepSeek-v3: $p = 0.038$**
					**Claude-3.5 vs DeepSeek-r1: $p = 0.009$**
					Claude-3.5 vs Claude-4: $p = 0.084$
					Claude-3.5 vs Claude-4 thinking: $p = 0.194$
					Moonshot-v1 vs ChatGLM-4: $p = 0.896$
					**Moonshot-v1 vs DeepSeek-v3: $p < 0.001$**
					**Moonshot-v1 vs DeepSeek-r1: $p < 0.001$**
					**Moonshot-v1 vs Claude-4: $p < 0.001$**
					**Moonshot-v1 vs Claude-4 thinking: $p = 0.009$**
					**ChatGLM-4 vs DeepSeek-v3: $p < 0.001$**
					**ChatGLM-4 vs DeepSeek-r1: $p < 0.001$**
					**ChatGLM-4 vs Claude-4: $p < 0.001$**
					**ChatGLM-4 vs Claude-4 thinking: $p = 0.008$**
					DeepSeek-v3 vs DeepSeek-r1: $p = 0.649$
					DeepSeek-v3 vs Claude-4: $p = 0.772$
					DeepSeek-v3 vs Claude-4 thinking: $p = 0.452$
					DeepSeek-r1 vs Claude-4: $p = 0.434$
					DeepSeek-r1 vs Claude-4 thinking: $p = 0.203$
					Claude-4 vs Claude-4 thinking: $p = 0.666$
Total	150	GPT-4o	4.00 (2.98-5.00) / 3.67 (1.34)	241.46 ($p < 0.001$)	**GPT-4o vs Claude-3.5: $p < 0.001$**
		Claude-3.5	2.70 (1.00-3.80) / 2.62 (1.49)		**GPT-4o vs Moonshot-v1: $p < 0.001$**
		Moonshot-v1	2.20 (1.00-3.03) / 2.42 (1.36)		**GPT-4o vs ChatGLM-4: $p < 0.001$**

			ChatGLM-4	3.00 (1.60-4.00) / 2.87 (1.36)		**GPT-4o vs DeepSeek-v3: $p < 0.001$**
			DeepSeek-v3	5.00 (4.00-5.00) / 4.27 (1.15)		**GPT-4o vs DeepSeek-r1: $p = 0.002$**
			DeepSeek-r1	5.00 (3.70-5.00) / 4.18 (1.16)		**GPT-4o vs Claude-4: $p = 0.002$**
			Claude-4	3.00 (1.00-4.16) / 3.02 (1.50)		**GPT-4o vs Claude-4 thinking: $p < 0.001$**
			Claude-4 thinking	2.85 (1.00-3.80) / 2.67 (1.55)		Claude-3.5 vs Moonshot-v1: $p = 0.278$
						Claude-3.5 vs ChatGLM-4: $p = 0.314$
						**Claude-3.5 vs DeepSeek-v3: $p < 0.001$**
						**Claude-3.5 vs DeepSeek-r1: $p < 0.001$**
						**Claude-3.5 vs Claude-4: $p = 0.034$**
						Claude-3.5 vs Claude-4 thinking: $p = 0.676$
						**Moonshot-v1 vs ChatGLM-4: $p = 0.032$**
						**Moonshot-v1 vs DeepSeek-v3: $p < 0.001$**
						**Moonshot-v1 vs DeepSeek-r1: $p < 0.001$**
						**Moonshot-v1 vs Claude-4: $p = 0.002$**
						Moonshot-v1 vs Claude-4 thinking: $p = 0.132$
						**ChatGLM-4 vs DeepSeek-v3: $p < 0.001$**
						**ChatGLM-4 vs DeepSeek-r1: $p < 0.001$**
						ChatGLM-4 vs Claude-4: $p = 0.282$
						ChatGLM-4 vs Claude-4 thinking: $p = 0.547$
						DeepSeek-v3 vs DeepSeek-r1: $p = 0.577$
						**DeepSeek-v3 vs Claude-4: $p < 0.001$**
						**DeepSeek-v3 vs Claude-4 thinking: $p < 0.001$**
						**DeepSeek-r1 vs Claude-4: $p < 0.001$**
						**DeepSeek-r1 vs Claude-4 thinking: $p < 0.001$**
						Claude-4 vs Claude-4 thinking: $p = 0.089$
Chinese	MDD	40	GPT-4o	5.00 (1.00-5.00) / 3.90 (1.81)	13.09 ($p = 0.070$)	/
			Claude-3.5	5.00 (1.00-5.00) / 3.40 (1.93)		
			Moonshot-v1	5.00 (1.00-5.00) / 3.15 (1.99)		
			ChatGLM-4	5.00 (3.50-5.00) / 4.05 (1.69)		
			DeepSeek-v3	5.00 (3.00-5.00) / 4.10 (1.43)		

		DeepSeek-r1	5.00 (3.00-5.00) / 4.15 (1.49)		
		Claude-4	5.00 (3.00-5.00) / 3.75 (1.55)		
		Claude-4 thinking	3.00 (3.00-5.00) / 3.50 (1.49)		
SPC	80	GPT-4o	4.30 (3.60-5.00) / 4.25 (0.79)	144.65 ($p < 0.001$)	**GPT-4o vs Claude-3.5: *p* = 0.027**
		Claude-3.5	4.30 (2.60-5.00) / 3.66 (1.27)		**GPT-4o vs Moonshot-v1: *p* < 0.001**
		Moonshot-v1	3.05 (2.20-4.10) / 3.07 (1.17)		GPT-4o vs ChatGLM-4: *p* = 0.498
		ChatGLM-4	4.30 (3.60-5.00) / 4.09 (0.88)		**GPT-4o vs DeepSeek-v3: *p* = 0.002**
		DeepSeek-v3	5.00 (4.70-5.00) / 4.62 (0.75)		**GPT-4o vs DeepSeek-r1: *p* = 0.039**
		DeepSeek-r1	5.00 (4.30-5.00) / 4.43 (1.01)		GPT-4o vs Claude-4: *p* = 0.818
		Claude-4	4.55 (3.33-5.00) / 3.96 (1.33)		**GPT-4o vs Claude-4 thinking: *p* < 0.001**
		Claude-4 thinking	3.10 (1.30-4.10) / 2.90 (1.47)		**Claude-3.5 vs Moonshot-v1: *p* = 0.003**
					Claude-3.5 vs ChatGLM-4: *p* = 0.141
					**Claude-3.5 vs DeepSeek-v3: *p* < 0.001**
					**Claude-3.5 vs DeepSeek-r1: *p* < 0.001**
					**Claude-3.5 vs Claude-4: *p* = 0.049**
					**Claude-3.5 vs Claude-4 thinking: *p* = 0.002**
					**Moonshot-v1 vs ChatGLM-4: *p* < 0.001**
					**Moonshot-v1 vs DeepSeek-v3: *p* < 0.001**
					**Moonshot-v1 vs DeepSeek-r1: *p* < 0.001**
					**Moonshot-v1 vs Claude-4: *p* < 0.001**
					Moonshot-v1 vs Claude-4 thinking: *p* = 0.928
					**ChatGLM-4 vs DeepSeek-v3: *p* < 0.001**
					**ChatGLM-4 vs DeepSeek-r1: *p* = 0.005**
					ChatGLM-4 vs Claude-4: *p* = 0.667
					**ChatGLM-4 vs Claude-4 thinking: *p* < 0.001**
					DeepSeek-v3 vs DeepSeek-r1: *p* = 0.364
					**DeepSeek-v3 vs Claude-4: *p* = 0.002**
					**DeepSeek-v3 vs Claude-4 thinking: *p* < 0.001**
					**DeepSeek-r1 vs Claude-4: *p* = 0.021**
					**DeepSeek-r1 vs Claude-4 thinking: *p* < 0.001**

					**Claude-4 vs Claude-4 thinking: $p < 0.001$**
CTA	30	GPT-4o	4.00 (3.00-5.00) / 3.80 (1.06)	51.85 ($p < 0.001$)	GPT-4o vs Claude-3.5: $p = 0.606$
		Claude-3.5	3.00 (3.00-5.00) / 3.57 (1.07)		GPT-4o vs Moonshot-v1: $p = 0.638$
		Moonshot-v1	3.00 (3.00-5.00) / 3.67 (0.96)		GPT-4o vs ChatGLM-4: $p = 0.988$
		ChatGLM-4	4.00 (3.00-5.00) / 3.77 (1.07)		**GPT-4o vs DeepSeek-v3: $p < 0.001$**
		DeepSeek-v3	5.00 (4.75-5.00) / 4.70 (0.57)		**GPT-4o vs DeepSeek-r1: $p < 0.001$**
		DeepSeek-r1	5.00 (4.75-5.00) / 4.70 (0.36)		**GPT-4o vs Claude-4: $p = 0.011$**
		Claude-4	5.00 (4.00-5.00) / 4.50 (0.86)		**GPT-4o vs Claude-4 thinking: $p = 0.009$**
		Claude-4 thinking	5.00 (4.00-5.00) / 4.53 (0.73)		Claude-3.5 vs Moonshot-v1: $p = 0.950$
					Claude-3.5 vs ChatGLM-4: $p = 0.606$
					**Claude-3.5 vs DeepSeek-v3: $p < 0.001$**
					**Claude-3.5 vs DeepSeek-r1: $p < 0.001$**
					**Claude-3.5 vs Claude-4: $p < 0.001$**
					**Claude-3.5 vs Claude-4 thinking: $p < 0.001$**
					Moonshot-v1 vs ChatGLM-4: $p = 0.693$
					**Moonshot-v1 vs DeepSeek-v3: $p < 0.001$**
					**Moonshot-v1 vs DeepSeek-r1: $p < 0.001$**
					**Moonshot-v1 vs Claude-4: $p = 0.002$**
					**Moonshot-v1 vs Claude-4 thinking: $p = 0.002$**
					**ChatGLM-4 vs DeepSeek-v3: $p < 0.001$**
					**ChatGLM-4 vs DeepSeek-r1: $p < 0.001$**
					**ChatGLM-4 vs Claude-4: $p = 0.008$**
					**ChatGLM-4 vs Claude-4 thinking: $p = 0.008$**
					DeepSeek-v3 vs DeepSeek-r1: $p = 0.999$
					DeepSeek-v3 vs Claude-4: $p = 0.606$
					DeepSeek-v3 vs Claude-4 thinking: $p = 0.606$
					DeepSeek-r1 vs Claude-4: $p = 0.606$
					DeepSeek-r1 vs Claude-4 thinking: $p = 0.606$
					Claude-4 vs Claude-4 thinking: $p = 0.999$
Total	150	GPT-4o	4.30 (3.60-5.00) / 4.07 (1.20)	135.49 ($p < 0.001$)	**GPT-4o vs Claude-3.5: $p = 0.007$**

Claude-3.5	4.15 (2.40-5.00) / 3.57 (1.44)	**GPT-4o vs Moonshot-v1: $p < 0.001$**
Moonshot-v1	3.05 (1.98-4.70) / 3.21 (1.42)	GPT-4o vs ChatGLM-4: $p = 0.729$
ChatGLM-4	4.30 (3.10-5.00) / 4.02 (1.19)	**GPT-4o vs DeepSeek-v3: $p < 0.001$**
DeepSeek-v3	5.00 (4.30-5.00) / 4.50 (0.98)	**GPT-4o vs DeepSeek-r1: $p = 0.005$**
DeepSeek-r1	5.00 (4.30-5.00) / 4.41 (1.11)	GPT-4o vs Claude-4: $p = 0.747$
Claude-4	5.00 (3.00-5.00) / 4.01 (1.34)	**GPT-4o vs Claude-4 thinking: $p < 0.001$**
Claude-4 thinking	3.80 (2.28-5.00) / 3.39 (1.49)	**Claude-3.5 vs Moonshot-v1: $p = 0.022$**
		**Claude-3.5 vs ChatGLM-4: $p = 0.021$**
		**Claude-3.5 vs DeepSeek-v3: $p < 0.001$**
		**Claude-3.5 vs DeepSeek-r1: $p < 0.001$**
		**Claude-3.5 vs Claude-4: $p = 0.003$**
		Claude-3.5 vs Claude-4 thinking: $p = 0.347$
		**Moonshot-v1 vs ChatGLM-4: $p < 0.001$**
		**Moonshot-v1 vs DeepSeek-v3: $p < 0.001$**
		**Moonshot-v1 vs DeepSeek-r1: $p < 0.001$**
		**Moonshot-v1 vs Claude-4: $p < 0.001$**
		Moonshot-v1 vs Claude-4 thinking: $p = 0.217$
		**ChatGLM-4 vs DeepSeek-v3: $p < 0.001$**
		**ChatGLM-4 vs DeepSeek-r1: $p = 0.002$**
		ChatGLM-4 vs Claude-4: $p = 0.540$
		**ChatGLM-4 vs Claude-4 thinking: $p = 0.002$**
		DeepSeek-v3 vs DeepSeek-r1: $p = 0.624$
		**DeepSeek-v3 vs Claude-4: $p = 0.002$**
		**DeepSeek-v3 vs Claude-4 thinking: $p < 0.001$**
		**DeepSeek-r1 vs Claude-4: $p = 0.010$**
		**DeepSeek-r1 vs Claude-4 thinking: $p < 0.001$**
		**Claude-4 vs Claude-4 thinking: $p < 0.001$**

N, Number of questions; LLMs, large language models; IQR, Interquartile Range; SD, Standard Deviation; K-W H, Kruskal-Wallis H test statistic; CM: Chinese Medicine; MDD, Medication based on Differential Diagnosis questions; SPC, Specific Prescription Consultation questions; CTA, CM Theory Analysis questions.

* Bold values indicate statistically significant differences ($p < 0.05$).

Supplementary Table S5. Comparison of accuracy scores between English and Chinese responses for each LLM

LLMs (N = 150)	English<br>Median (IQR) / Mean (SD)	Chinese<br>Median (IQR) / Mean (SD)	Z-score*	p-value
GPT-4o	4.00 (2.98-5.00) / 3.67 (1.34)	4.30 (3.60-5.00) / 4.07 (1.20)	-3.784	< 0.001
Claude-3.5	2.70 (1.00-3.80) / 2.62 (1.49)	4.15 (2.40-5.00) / 3.57 (1.44)	-6.376	< 0.001
Moonshot-v1	2.20 (1.00-3.03) / 2.42 (1.36)	3.05 (1.98-4.70) / 3.21 (1.42)	-6.118	< 0.001
ChatGLM-4	3.00 (1.60-4.00) / 2.87 (1.36)	4.30 (3.10-5.00) / 4.02 (1.19)	-7.846	< 0.001
DeepSeek-v3	5.00 (4.00-5.00) / 4.27 (1.15)	5.00 (4.30-5.00) / 4.50 (0.98)	-2.7	0.007
DeepSeek-r1	5.00 (3.70-5.00) / 4.18 (1.16)	5.00 (4.30-5.00) / 4.41 (1.11)	-3.004	0.003
Claude-4	3.00 (1.00-4.16) / 3.02 (1.50)	5.00 (3.00-5.00) / 4.01 (1.34)	-6.717	< 0.001
Claude-4 thinking	2.85 (1.00-3.80) / 2.67 (1.55)	3.80 (2.28-5.00) / 3.39 (1.49)	-5.418	< 0.001

LLMs, large language models; N, Number of questions.

* Wilcoxon signed-rank test was used to compare accuracy scores between English and Chinese responses for each LLM. Negative Z-scores indicate higher accuracy in Chinese responses.

Supplementary Table S6. Comparison of 8 LLMs Performance on MDD Questions With and Without Rationale Requirements

Languages	Rationale not required<br>Median (IQR) / Mean (SD)	Rationale required<br>Median (IQR) / Mean (SD)	Z-score*	p-value
English (N = 320)	3.00 (1.00-5.00) / 3.11 (1.82)	5.00 (1.00-1.00) / 3.24 (1.92)	-1.053	0.292
Chinese (N = 320)	5.00 (3.00-5.00) / 3.75 (1.70)	5.00 (5.00-5.00) / 4.02 (1.72)	-2.254	0.024
Total (N = 640)	5.00 (1.00-5.00) / 3.43 (1.79)	5.00 (1.00-5.00) / 3.63 (1.87)	-2.355	0.019

LLMs, large language models; MDD, Medication based on Differential Diagnosis; N, Number of questions.

Supplementary Table S7. Readability of the responses of the LLMs

Language	LLMs	Number of answers	Readability Score Mean (SD)	Response length (words)			Response length (characters)		
				Mean (SD)	Minimum	Maximum	Mean (SD)	Minimum	Maximum
English	GPT-4o	150	39.33 (12.71)	172.85 (97.09)	31	564	1209.57 (696.96)	276	4235
	Claude-3.5 sonnet	150	30.52 (14.36)	175.21 (61.80)	53	405	1273.87 (438.94)	428	2804
	Moonshot-v1	150	36.21 (9.03)	221.13 (80.48)	50	406	1488.89 (520.42)	346	2675
	ChatGLM-4	150	33.63 (13.47)	300.07 (122.81)	3	509	2039.19 (824.70)	48	3614
	DeepSeek-v3	150	40.10 (13.27)	300.68 (93.02)	71	544	3080.19 (1340.70)	516	4974
	DeepSeek-r1	150	36.43 (10.63)	593.64 (144.69)	139	834	3767.65 (809.73)	978	4974
	Claude-4 sonnet	150	24.53 (16.28)	167.82 (41.29)	68	358	1302.03 (320.03)	527	2231
	Claude-4 sonnet thinking	150	20.66 (14.59)	165.54 (28.85)	60	268	1291.29 (232.53)	554	2034
	Total	1200	32.68 (14.71)	/	/	/	/	/	/
Chinese	GPT-4o	150	11.26 (1.57)	249.10 (124.39)	51	497	255.75 (125.71)	56	508
	Claude-3.5 sonnet	150	11.36 (1.07)	305.57 (114.02)	116	575	316.00 (116.24)	122	585
	Moonshot v1	150	11.78 (1.01)	375.17 (122.51)	107	731	383.53 (124.89)	113	752
	ChatGLM-4	150	10.99 (2.16)	333.30 (176.03)	5	748	341.77 (180.10)	5	767
	DeepSeek-v3	150	12.24 (1.45)	581.44 (264.43)	126	1374	646.63 (268.70)	151	1374
	DeepSeek-r1	150	12.70 (1.69)	139.33 (209.10)	525	1715	1476.33 (251.07)	565	1972
	Claude-4 sonnet	150	11.74 (2.46)	345.38 (89.57)	151	579	381.09 (92.00)	174	579
	Claude-4 sonnet thinking	150	11.87 (2.22)	313.99 (93.20)	74	640	342.65 (93.36)	95	640
	Total	1200	11.74 (1.84)	/	/	/	/	/	/

LLMs, large language models.

Supplementary Table S8. Examples of reasons contributing to score deduction in llm outputs

Type	Questions	Answers （clinical practice guideline）	Content of Large Language Models' Responses	Reasons contributing to score deduction
MDD	For the treatment of pediatric viral pneumonia with a syndrome of phlegm-heat obstructing the lungs, what Chinese medicine formulas should be used? Could you recommend several commonly used CM prescriptions, and just tell me the names of the medicines?	Wu Hu Tang; Ting Li Da Zao Xie Fei Tang	For pediatric viral pneumonia with a syndrome of phlegm-heat obstructing the lungs, Chinese medicine (CM) commonly utilizes formulas aimed at clearing heat, resolving phlegm, and easing breathing. Here are some commonly used CM prescriptions:<br>1.Qing Qi Hua Tan Wan (Clear Qi and Transform Phlegm Pill)<br>2.Ma Xing Shi Gan Tang (Ephedra, Apricot Kernel, Gypsum, and Licorice Decoction)<br>3.Xiao Xian Xiong Tang (Minor Sinking into the Chest Decoction)<br>4.Er Chen Tang (Two Aged Decoction)<br>5.Zhi Sou San (Stop Coughing Powder)<br>These formulas are selected based on the specific symptoms and the overall condition of the patient. (GPT-4o)	◆Inconsistent with clinical practice guidelines
SPC	For the treatment of endometriosis pain with the pattern of qi stagnation and blood stasis, do you recommend using the Chinese patent medicine Dan ’ e Fukang Jiao Gao to treat this condition? What are the specific usage instructions and dosage? Please answer whether you recommend it and respond to my questions.	For the condition of pain due to endometriosis with the syndrome of qi stagnation and blood stasis, it is recommended to use Dan'e Fukang Decoction alone or in combination with Western medicine for treatment (Evidence Level: C, Strong Recommendation). The composition of the medicine includes: miltiorrhiza, Curcuma zedoaria, Bupleurum chinense, Notoginseng, Paeonia lactiflora, Angelica sinensis, Curcuma zedoaria	I do not actually recommend specific medical treatments or provide dosage instructions. Chinese medicine can have complex effects and potential interactions, so it's important to consult with a qualified practitioner for personalized advice on using remedies like Dan'e Fukang Jiao Gao for endometriosis. They can evaluate your individual condition and recommend an appropriate treatment plan. For any medical concerns, I encourage speaking with a licensed healthcare provider who can give proper medical guidance. (Claude-3.5 sonnet)	◆Shows no attention to the patient's specific issues and symptoms<br>◆Provides no explanation of the basic effects of Dan'e Fukang capsules<br>◆Overemphasizes liability disclaimers<br>◆Fails to demonstrate understanding of the patient's concerns

(three-edged), Cyperi Rhizoma, Corydalis yanhusuo, and Glycyrrhiza uralensis. Usage and dosage: Oral administration, 10-15 grams per dose, twice a day. Start 10-15 days before menstruation and continue for 10-15 days as one course of treatment, without stopping during the menstrual period. Contraindications and precautions: Contraindications: Do not use during pregnancy, for diabetic patients, or for those allergic to this product. Precautions: Use with caution for people with allergic constitutions. If there is a desire for fertility, it should be taken under the guidance of a physician. If excessive menstrual bleeding or worsening of symptoms occurs during the medication period, please consult a doctor promptly. To avoid gastrointestinal discomfort, it is advisable to take it after meals. Do not use in conjunction with Mirabilite, Senna, seaweed, Euphorbia pekinensis, Euphorbia helioscopia, Euphorbia kansui, or Glaucium flavum. Adding an appropriate amount of honey can improve the taste. One RCT study (400 patients, 3 months) showed that the effectiveness of Dan'e Fukang Decoction in treating dysmenorrhea due to endometriosis was not significantly different from that of gestrinone [RR = 0.98, 95% CI (0.91, 1.05)]

◆Offers no substantive advice or next steps

	(Evidence Level: B). Safety: Seven RCT studies (1269 patients, 3 months of treatment) showed that the incidence of adverse reactions of Dan'e Fukang Decoction was lower than that of gestrinone [RR = 0.12, 95% CI (0.08, 0.19), I^2 = 84%] (Evidence Level: D).		
For the treatment of flooding and leaking with the pattern of spleen deficiency, do you recommend using the Chinese herbal formula Guchong Decoction? What specific medicinal ingredients does it contain? Please answer whether you recommend it and answer my question.	For uterine bleeding during the bleeding period with spleen deficiency syndrome, the recommended prescription is Guchong Decoction (Level A evidence; Recommendation Strength: Strong. Expert consensus: 95%). The basic ingredients include Atractylodes macrocephala (Bai Zhu), Astragalus membranaceus (Huang Qi), Fossilized dragon bones (Long Gu), Ostrea gigas (Mu Li), Cornus officinalis (Shan Yu Rou), Paeonia lactiflora Pall. var. trichocarpa (Bai Shao), Sepiella maindroni (Hai Piao Xiao), Rubia cordifolia (Qian Cao), Rhus chinensis (Zong Lu Tan), and Gallnut (Wu Bei Zi).	Chinese Medicine (CM) has a long history of treating various health conditions, including those related to spleen deficiency. However, it's important to note that the use of any herbal formula should be guided by a qualified CM practitioner who can take into account the individual's specific symptoms, overall health, and medical history.<br>Guchong Decoction (also known as Gui Zhi Fu Ling Tang) is a classic CM formula that is traditionally used to treat conditions characterized by spleen deficiency with symptoms like fatigue, lack of appetite, and loose stools. The formula is designed to strengthen the spleen and harmonize the digestive system.<br>The typical ingredients in Guchong Decoction include:<br>1.Cinnamomum cassia (Rou Gui) - to warm the interior and strengthen the yang.<br>2.Poria cocos (Fu Ling) - to strengthen the spleen and eliminate dampness.<br>3.Atractylodes macrocephala (Bai Zhu) - to strengthen the spleen and dry dampness.<br>4.Zingiber officinale (Gan Jiang) - to warm the middle and support digestion.<br>5.Jujuba fruit (Da Zao) - to nourish the spleen and calm	◆Inconsistent with clinical practice guidelines

			the spirit.<br>6.Glycyrrhiza uralensis (Gan Cao) - to harmonize the actions of the other herbs and to support the spleen.<br>It's important to consult with a CM practitioner before starting any herbal treatment, as they can provide a personalized assessment and recommend the appropriate treatment based on your specific needs. Self-medicating without professional advice can lead to inappropriate treatment and potential side effects. (Moonshot v1)	
CTA	It seems that there is no specific disease name for acute tracheobronchitis in ancient Chinese medicine. Generally, under what category is acute tracheobronchitis considered in Chinese medicine?	Acute tracheobronchitis falls within the category of "cough" in Chinese Medicine (CM).	In Chinese medicine (CM), acute tracheobronchitis does not have a specific disease name but can be categorized under the broader concept of “wind-cold” or “wind-heat” syndromes depending on the symptoms presented.<br>1.Wind-Cold Syndrome: This type of syndrome typically presents with symptoms such as chills, fever, headache, body aches, and coughing up clear mucus. In CM, this condition is often treated by expelling wind and cold from the body using herbs like ephedra, cinnamon twig, and ginger.<br>2.Wind-Heat Syndrome: This syndrome usually involves symptoms like high fever, sweating, thirst, sore throat, and yellow phlegm. Treatment may involve clearing heat and expelling wind using herbs like honeysuckle, forsythia, and mint.<br>The treatment approach in CM would also include dietary adjustments, acupuncture, moxibustion, cupping therapy, and possibly herbal formulas tailored to the individual’s constitution and specific symptoms. It’s important to note that while these treatments are part of CM practice, they	◆The Misinterpretation of CM Categories as Syndromes (Patterns)

			should be used alongside modern medical care when dealing with acute conditions like tracheobronchitis. (ChatGLM-4)

CM: Chinese Medicine; MDD, Medication based on Differential Diagnosis questions; SPC, Specific Prescription Consultation questions; CTA, CM Theory Analysis questions.
